# Visual Performance of Psychological Factors in Interior Design Under the Background of Artificial Intelligence

**DOI:** 10.3389/fpsyg.2022.941196

**Published:** 2022-07-28

**Authors:** Yunkai Xu, TianTian Yu

**Affiliations:** ^1^School of Textile Apparel and Design, Changshu Institute of Technology, Suzhou, China; ^2^Wenzhou Polytechnic University, Wenzhou, China; ^3^School of Housing, University of Science, Penang, Malaysia

**Keywords:** artificial intelligence, interior design, optical illusion, psychological factors, visual performance

## Abstract

Sensation (the reflection of past experience in the mind) is the reflection of the brain on the individual attributes of objective things that directly act on the sense organs. Feeling is the most elementary cognitive process and the simplest psychological phenomenon. Vision is a kind of sense, and sense is produced by objective things acting on the sense organs. But at present, it is rare to analyze interior design exhibition from the perspective of visual psychology, an emerging science, as an interdisciplinary attempt, only in interior design research. Therefore, the study of sensory process should start from its external stimuli, in order to first understand how it acts on the sensory organs to produce sensory phenomena. This paper mainly studies the visual performance of psychological factors in interior design under the background of artificial intelligence. This paper proposes a K-means clustering algorithm and a localization algorithm fused with visual and inertial navigation. The distance thresholds corresponding to the SIFT feature descriptors of threshold T1, 128D, 96D, 64D, and 32D are 170, 160, 150, and 90, respectively. This verifies that the candidate image with the highest number of matching points is considered the best matching image.

## Introduction

How to make the design form and design method of interior design closer to the audience’s thinking from the perspective of human visual psychology, so that the content delivered by interior design can take into account the accuracy and artistry. According to different needs of people, sometimes the room needs to be warm and comfortable; sometimes it needs to be solemn and dignified; and sometimes it needs to be relaxed. When designers do interior design, they always try to make the room transparent, spacious, and bright. But the problem we face is that the plane of the room is fixed, the height of the room is also fixed, and the position and size of the windows in the room are also fixed. The hallucination image is not vivid and vivid, and it occurs in the patient’s subjective space, such as the brain and the body. Hallucinations are not obtained through the sense organs. For example, if you hear a voice in your stomach, you can see a human figure in your mind without your own eyes. This all needs to be changed through the technique of optical illusion decoration.

In China, many rooms only face north. In today’s crowded social housing conditions, there are many small units. The population density is high, and people have more and more items. Designers need to decorate the room to make it less crowded. Interior design needs to use optical illusions to cover up the shortcomings of the room, or learn from each other’s strengths. Through comprehensive analysis, it can meet the different needs of today’s people for indoors. According to the current national conditions, such as apartment type, space size, etc., are fixed, it is necessary to consider how to make the narrow space appear spacious, bright, low, and depressed room. As for how to increase the permeability of a relatively closed room, etc., it can be improved by means of optical illusions. Although the illusion has been discovered for a long time, its application in interior design is relatively new at home and abroad.

The innovation of this paper is that the perfect explanation makes use of optical illusion effects. It responds perfectly to the visual analysis of interior design, and utilizes advanced technologies and instruments for indoor positioning to perform experimental measurements to verify conjectures. In this paper, the K-means clustering algorithm is further improved and utilized. Interior design is closely related to people’s visual senses and psychological characteristics. The human eye has a focus and sequence when viewing things. This visual psychological habit of people will guide our visual process and psychological thinking, so we are carrying out In interior design, text, graphics, and colors should be arranged according to people’s visual and psychological habits, and at the same time to achieve the esthetic sense of formal art and people’s emotional acceptance, so as to better transmit the design content.

## Related Work

Interior design is to create an indoor environment with reasonable functions, comfortable and beautiful, and meet people’s material and spiritual needs according to the nature of use of the building, the environment, and the corresponding standards, using material technical means and architectural design principles. Logos in different regions may have different characteristics that affect the likes of the logo. Zhu et al. (2017) mainly discussed how logo design features affect consumer responses based on visual representation. To understand the extent to which indoor environmental factors influence the creation of an optimal rehabilitation environment, Mahmood and Tayib (2019) proposed that contemporary rehabilitation environments may be affected by the visual effects of indoor environmental quality. Celadyn (2018) discussed the issues that the modification of contemporary interior design strategies and methods should be introduced into the interior design model oriented by environmental responsibility. On the basis of previous research results, Zhang and Zheng (2017) explored the ideas and methods of realizing realistic indoor scenes based on the interactive real-time rendering of the open-source project MNRT. He used NVIDIA’s powerful application engine, ray tracing engine Opti X, scene management engine SceniX, and PhysX engine to design the commercial design of interior design software. Aiming at the significant and abundant color difference between regions in interior design, Fang et al. (2017) proposed a color transfer algorithm for interior design based on region matching based on topology information. He first introduced the segmentation of interior design images, and then proposed to calculate the topological information of each region to determine the matching relationship between regions. This, in turn, improves the accuracy of color transfer between images for enhanced visual effects. Celadyn (2017) analyzed the options for adapting an interior design education model to the principles of environmental sustainability in the creation of interior environments. This is because the current curriculum of most interior design schools is established according to the traditional design scheme. Ju and Yang (2021) research developed and applied a service-learning curriculum. The program combines university courses with housing and interior design in the local community. These studies have better explained the importance of the central psychological factors of interior design to visual performance, but they are still not comprehensive enough and not innovative enough.

## The Visual Expression Algorithm of Psychological Factors in Interior Design

### K-means Clustering Algorithm

Artificial intelligence is the study of making computers to simulate certain thinking processes and intelligent behaviors (such as learning, reasoning, thinking, planning, etc.) and enables computers to achieve higher-level applications. The K-means algorithm is an unsupervised method that was formally mentioned in the early 1960s. The basic idea of the algorithm is to classify the m data types in the data set according to the preset threshold rules, and then divide them into p types. This satisfies the condition that the similarity between the same type of data after classification is high, while the similarity between different types of data is relatively low (Chang and Yang, 2020). It assumes that the p cluster centers of the dataset *w* = (a_1_, a_2_, a_3_,…a_m_) that need to be clustered are (b_1_, b_2_,…b_m_). By default, it uses the Euclidean distance to measure the distance between two samples.


(1) $$d\left(a_{i},a_{j}\right)={\left(A_{i1}-A_{j1}\right)}^{2}+{\left(A_{i2}-A_{j2}\right)}^{2}+\ldots \ldots +{\left(A_{im}-A_{jm}\right)}^{2}$$

Among them, *a_i_*, *a_j_* represents two m-dimensional data objects (Caulfield, 2018).

The average algorithm for all samples is as follows.


(2) $$M\mathrm{eandist}\;\left(W\right)=\frac{2}{m\left(m-1\right)}\times \overset{m}{\underset{i\neq j}{\sum }}d\left(a_{i},a_{j}\right)$$

The most commonly used objective function is the squared error criterion function, which is defined as.


(3) $$Q=\overset{P}{\underset{I=1}{\sum }}\underset{J\in m_{i}}{\sum }||a_{j}-c_{i}|{|}^{2}$$

In the above formula, *m_i_* represents the ith cluster set and *c_i_* represents the center of the ith cluster. Euclidean distance is also called Euclidean distance. In n-dimensional space, the length of the shortest line is its Euclidean distance. It is a commonly used definition of distance, which is the true distance between two points in m-dimensional space. *Q* represents the sum of squares of the distances between all the data in the dataset and their respective cluster centers. The distance here refers to the Euclidean distance.

The specific steps of K-means clustering are as follows: (1) It first pre-determines the number of clusters *k* according to actual needs, and then selects *k* data from the given sample data. This selection is random and it is used as the initial clustering point. (2) It calculates the distances between all remaining data objects and the selected K cluster center points, and classifies them into the corresponding categories with the smallest similarity. (3) For each generated class, it calculates the average sum of all data in that class and takes it as the new cluster center point (Wallace, 2019). (4) It judges whether the iteration can be completed according to the requirements of the iteration completion. It assumes that the condition can be completed iteratively. The conditions for the end of the iteration of the K-means algorithm:

1. The cluster no longer changes in any way.
2. The difference between the objective function values of the two iterations before and after is lower than the pre-computed threshold.
3. It iterates more than the previously specified number of times. And as long as any one of the above three conditions is satisfied, the iteration can end. If either condition (1) or (2) is satisfied, the clustering is convergent. The K-means algorithm is really fast for big data processing, but its shortcomings are also obvious. The algorithm is very sensitive to the selected cluster center points; however, the results are indeed greatly affected by this. At first, it selects different initial random seed points, which may eventually lead to different clustering results, or even farther apart.

### SVM Classifier

In machine learning, support vector machines (SVMs, also support vector networks) are supervised learning models related to related learning algorithms that can analyze data, identify patterns, and use for classification and regression analysis. Vector machines were formally proposed in the last century as a machine learning method for classification (Shubha and Meera, 2019). The core of the algorithm is to exploit the principle of risk minimization. It aims to find a hyperplane that separates all the different data and thus achieves the highest classification accuracy (Grobelny et al., 2021). It assumes that the set of samples to be trained is D, as shown in Formula (4). It contains n features, and the classification hyperplane is recorded as Formula (5).


(4) $$R={\left\{\left(x_{i},y_{i}\right)\left|x_{i}\in p^{d},y_{i}\in \left\{-1,1\right\}\right.\right\}}_{i=1}^{m}$$

(5) wx+t=0

For data that need to be classified, it is usually divided into two different cases according to whether it is linear or not.

For the first ideal case, the classification interval is usually set to 2/||w||, and there are constraints shown in Formula (6) below (Jain et al., 2022).


(6) $$y_{i}\left(wx_{i}+t\right)\geq 1,1\leq i\leq m$$

According to the constraints of Formula (6) above, the problem of constructing the optimal hyperplane is transformed into the solution problem shown in the following Formula (7):


(7) $$\min\alpha \left(w\right)=||w|{|}^{2}/2$$

With the introduction of the Lagrangian operator, Formula (7) can be transformed into its dual problem, which can be obtained as:


(8) $$\{\begin{array}{l} \max\left(\beta \right)=\overset{i=1}{\underset{m}{\sum^{\text{​}}}}\beta_{i}-\frac{1}{2}\overset{i=1}{\underset{m}{\sum^{\text{​}}}}\beta_{i}\beta_{j}y_{i}y_{j}x_{i}x_{j}\\ s.b.\beta_{i}\geq 0,\overset{i=1}{\underset{m}{\sum^{\text{​}}}}\beta_{i}y_{i}=0,1\leq i\leq m,1\leq j\leq m \end{array}$$

Combining the above formulas, the optimal solutions of the hyperplanes such as Formulas (9) and (10) can be obtained.


(9) $$w=\overset{m}{\underset{i=1}{\sum }}\beta_{i}y_{i}x_{i}$$

(10) $$t=y-wx_{i}$$

Finally, the optimal classification function can be obtained by solving the Formula (11).


(11) $$f\left(x\right)=\overset{m}{\underset{i=1,j=1}{\sum }}\beta_{i}\beta_{j}x_{i}x_{j}+t$$

In view of the fact that the vast majority of sample data is linearly inseparable, if the linear SVM classification method is also used at this time, it is likely to lead to serious wrong classification results (Horton, 2017). In view of this situation, researchers at home and abroad have improved the SVM30, that is, it introduced the kernel function. In the face of linear inseparability, the way vector machines work is to first choose to complete the calculation in a low-dimensional space. It then uses the function of the kernel function to map the input feature space to a relatively high-dimensional spatial feature space, and finally realizes the high-dimensional feature space. The construction of the optimal separating hyperplane is done in the feature space. This thus completes the separation of non-linear data that is not easily separable on the plane itself.


(12) $$\max\left(\beta \right)=\overset{m}{\underset{i=1}{\sum }}\beta_{i}-\frac{1}{2}\overset{m}{\underset{i=1,j=1}{\sum }}\beta_{i}\beta_{j}y_{i}y_{j}K\left(x_{i}x_{j}\right)$$

*k* is the introduced kernel function.

The so-called radial basis function is a scalar function that is symmetrical along the radial direction. It is usually defined as a monotonic function of the Euclidean distance between any point *x* in space and a certain center xc. Gaussian radial basis kernel function:


(13) $$k\left(x\mathrm{,y}\right)=exl\left(-\frac{{\left|x-y\right|}^{2}}{2\varepsilon }\right)$$

It assumes that the choice of parameter ε determines the width of the radial basis function. Therefore, its selection has a great influence on the final classification result. This kernel function can be said to be the most widely used among the multiple kernel functions. It will have a better effect regardless of the size of the sample. And it also has the advantage that it has fewer parameters than the polynomial kernel function. Therefore, it can consider the preferred Gaussian kernel function without knowing the specific case of the sample. According to pattern recognition theory, linearly inseparable patterns in low-dimensional space may be linearly separable by non-linear mapping to high-dimensional feature space, but if this technique is directly used for classification or regression in high-dimensional space, there are certain forms and parameters of the linear mapping function, the dimension of the feature space, etc., and the biggest obstacle is the “curse of dimensionality” that exists in the operation of the high-dimensional feature space. The use of kernel function technology can effectively solve such problems.

For indoor scene classification algorithms, we generally use the two indicators of accuracy and running time to judge (Ponde et al., 2017). The confusion matrix of scene classification results based on bag-of-words model is shown in Table 1. The confusion matrix of scene classification results based on the spatial pyramid bag-of-words model is shown in Table 2.

**Table 1:** Confusion matrix of scene classification results for bag-of-words model.

Bedroom	0.90	0.00	0.00	0.10	0.00
Boardroom	0.00	0.80	0.15	0.05	0.00
Corridor	0.05	0.15	0.75	0.00	0.00
Kitchen	0.00	0.10	0.15	0.00	0.00
Living room	0.20	0.10	0.00	0.80	0.00
Stairway	0.05	0.05	0.10	0.00	0.90

**Table 2:** Confusion matrix of scene classification results for pyramid bag-of-words model.

Bedroom	1.00	0.00	0.10	0.10	0.05
Boardroom	0.00	0.70	0.15	0.05	0.00
Corridor	0.05	0.15	0.80	0.00	0.10
Kitchen	0.15	0.10	0.15	0.90	0.00
Living room	0.20	0.15	0.05	0.80	0.50
Stairway	0.05	0.05	0.10	0.00	0.90

The classification of the two scenes, Kitchen and Boardroom, is easy to be confused, and classification and recognition errors are prone to occur. Its error rate can even reach 30% (Resch, 2019). The accuracy rate does not meet the high-standard scene classification requirements. The classification accuracy results of all scene classification algorithms are shown in Table 3. The running times of all scene classification algorithms are shown in Table 4. In order to ensure the reliability of the algorithm, we simulate and run each algorithm for three times in the simulation experiment, and finally take the average value.

**Table 3:** Classification results for all categories of images.

	The first time (%)	The second time (%)	The third time (%)	Average value (%)
Classic bag-of-words model	77.5	75.8	78.3	77.2
Pyramid bag-of-words model	82.5	80.8	83.3	82.2
Pyramid that only improves the K-means algorithm bag-of-words model	85.8	84.2	85.8	85.2
The improved pyramid bag-of-words model in this paper	90.8	91.7	90.8	91.1

**Table 4:** Temporal statistics for classification of all classes of images.

	The first time (min)	The second time (min)	The third time (min)	Average value (min)
Classic bag-of-words model	10.5	10.1	11.5	11.7
Pyramid bag-of-words model	14.1	13.2	15.1	14.1
Pyramid that only improves the K-means algorithm bag-of-words model	17.6	16.8	18.2	17.5
The improved pyramid bag-of-words model in this paper	19.4	18.2	20.2	19.3

From the data in Table 3, it can be seen that the accuracy of the scene classification algorithm based on the spatial pyramid bag-of-words model is 5% higher than that of the bag-of-words classification algorithm without the SPM model. Its scene classification rate can reach more than 80% (Leung and Zhang, 2019). Combined with the K-means clustering algorithm rule of the roulette method, it is also improved by 3% compared with the original bag-of-words model classification algorithm based on the spatial pyramid. In addition, it can be seen that there is room for improvement in this paper. The bag-of-words model of the pyramid is also effectively optimized by the classification algorithm to improve the defects of the K-means clustering algorithm. In general, the scene classification accuracy of the improved algorithm in this paper is indeed improved compared with the original scene classification algorithm based on the spatial pyramid model. This can verify that the improved algorithm has the highest scene classification accuracy.

It can be seen from the data in Table 4 that the running time of the spatial pyramid bag-of-words model based on the K-means clustering algorithm combined with the roulette method differs by nearly 3 min from the running time of the algorithm. Overall, the scene classification algorithm improved by the bag-of-words model based on the spatial pyramid has the longest running time. It is almost twice as fast as the bag-of-words-based classification algorithm, but the overall time is different.

### Localization Algorithm Fusion of Vision and Inertial Navigation

The visual positioning and navigation technology is carried out on the basis of a series of work such as feature matching of images of a series of frames before and after. This results in that the accuracy of visual localization navigation depends to a large extent on whether the amount of features of the image is sufficiently accurate. When the features are scarce or there is a mismatch, the accuracy of the localization is prone to large errors (Jeamwatthanachai, 2019). In order to improve this situation, in this chapter, a positioning algorithm combining visual and inertial navigation is proposed by taking advantage of the advantage of the inertial measurement unit with high positioning accuracy in a very short time. The basic principle of visual automatic positioning technology is to transmit the collected physical image to the PLC image-processing system through the CCD brought by the machine equipment, calculate the offset position and angle through the image processing positioning software, and then feed it back to the external platform for motion control. The position correction function is completed by the precision servo drive. The accuracy can be manually set, and if the accuracy exceeds the range, the system will report an alarm if it cannot be completed.

The inertial coordinate system ($G_{i}x_{i}y_{i}z_{i}$ is denoted as the *i* system) refers to a coordinate system that has no acceleration and rotation relative to the rest of the universe, and there is no absolute inertial coordinate system. When the motion carrier is limited to the motion near the earth, if the orbital motion of the earth is ignored, the geocentric inertial coordinate system can be used as an ideal approximation of the inertial coordinate system. Any point w near the earth can be represented by three-dimensional coordinates P(x, y, z). Of course, any point on the earth’s surface can also be represented by the latitude and longitude coordinate system. It assumes that the latitude and longitude coordinates of any point P are expressed as P(ϕ, L), and the conversion relationship between the latitude and longitude coordinate system to the earth coordinate system can be expressed by a formula.


(14) $$\{\begin{array}{l} x=R_{M}\cos L\cos\phi \\ y=R_{M}\cos L\sin\phi \\ z=R_{M}{\left(1-v\right)}^{2}\sin L \end{array}$$

The conversion relationship between the earth and the latitude and longitude coordinate system is shown in Formula (15).


(15) $$\{\begin{array}{l} \phi =\arctan\frac{y}{x}\\ L=\arctan\left[\frac{1}{\left(1-v\right)}\times \frac{z}{x^{2}+y^{2}}\right] \end{array}$$

$R_{M}$ and *v* are the radius of curvature and flattening of the Earth, respectively.

Multiplication of quaternions is not commutative. Quaternions are, unambiguously, non-commutative extensions of complex numbers. If the set of quaternions is considered as a multi-dimensional real number space, the quaternion represents a four-dimensional space, which is a two-dimensional space relative to complex numbers. In the calculation of the change of position of inertial navigation and positioning by considering the idea of representing the rotation matrix through the quaternion, the core part is the quaternion differential formula. It now combines some inherent properties of quaternions to analyze its differential formula as follows:

It is assumed in advance that the coordinate system GXYZ at time d has a rotation relative to the original coordinate system $G_{i}x_{i}y_{i}z_{i}$. It assumes that this rotation is $G_{1}$, then formula $Q_{1}{}^{-1}$ can be obtained.


(16) $$R_{i}\left(d\right)=Q_{1}R\left(d\right)Q_{1}{}^{-1}$$

In Formula (16), *Q*_1_ is a quaternion representing rotation, and we get the form of rotation operator through *Q*_1_, that is, $Q_{1}Q_{1}{}^{-1}$. For the short-time variation range of *Δd*, due to the angular velocity w of the coordinate system Oxyz, the relative positions of the above two coordinate systems also change immediately. At this point, we see that the coordinate system $G_{i}x_{i}y_{i}z_{i}$ of Oxyz has changed to $Q_{2}$ for the previously fixed coordinate system. At this time, the formula can be obtained.


(17) $$R_{i}\left(d+\Delta d\right)=Q_{2}R\left(d+\Delta d\right)Q_{2}{}^{-1}$$

For a brief time range of *Δd*, the motion changes and the rotational form is now represented by $Q_{1}{}^{-1}Q_{2}$. Since we assume that the value of *Δd* is very small, the change of the angular rate S existing for the coordinate system is regarded as a relatively fixed constant value. It can then obtain the angular displacement of the dynamic system shown in the formula. At that time, it was mainly text-based image retrieval technology. Use text description to describe the characteristics of the image, such as the author, age, genre, size, etc., of the painting. After the 1990s, the image retrieval technology that analyzes and retrieves the content semantics of the image, such as the color, texture, and layout of the image, has emerged, that is, the content-based image retrieval technology. The improved bag-of-words model algorithm is its core algorithm.


(18) $$\Delta \phi =\left|S\right|\Delta d$$

In Formula (18), the value of s is reflected by the modulo |s| of *s*, but the direction of Δ1 still depends on the direction of *s*. It assumes a unit vector $\eta =\frac{s}{\left|s\right|}$ to obtain Formula (19):


(19) $$Q_{1}{}^{-1}Q_{2}=\cos\frac{\left|s\right|\Delta d}{2}+\eta \sin\frac{\left|s\right|\Delta d}{2}$$

From Formula (19), *Q*_2_ can be obtained after simple calculation, and *Q*_2_ can be expressed by Formula (20).


(20) $$Q_{2}=Q_{1}\left(\cos\frac{\left|s\right|\Delta d}{2}+\eta \sin\frac{\left|s\right|\Delta d}{2}\right.$$

It finally performs the derivation work for the quaternion *Q*, and the derivative of *Q* can be easily obtained as shown in Formula (21).


(21) $$Q^{\prime }\left(t\right)={\underset{\Delta d\to 0}{\lim}}_{\Delta d}^{\Delta d^{Q_{2}-Q_{1}}}={\underset{\Delta d\to 0}{\lim}}_{\Delta d}^{\Delta d^{Q_{1}}}\left(\cos\frac{\left|s\right|\Delta d}{2}+\eta \sin\frac{\left|s\right|\Delta d}{2}-1\right)$$

It firstly studies the theoretical part of inertial navigation. Aiming at the problem of the cumulative error of visual positioning, it proposes a visual positioning algorithm fused with inertial navigation by taking advantage of the high accuracy of inertial navigation in a short time. The core of the algorithm is to use the rotation matrix R and translation vector T of the IMU solution result as the initial value of the camera motion. It sets a threshold and removes the matching points that obviously do not satisfy the motion model between adjacent frames of the camera as outliers. Through the simulation experiments of two indoor scenes, it is compared and analyzed that the visual positioning algorithm of the integrated inertial navigation proposed in this paper has higher positioning accuracy and better effect than the visual positioning algorithm.

## Experimental Analysis on the Visual Performance of Design by Psychological Factors in Interior Design

### Experimental Method

**Figure 1:**
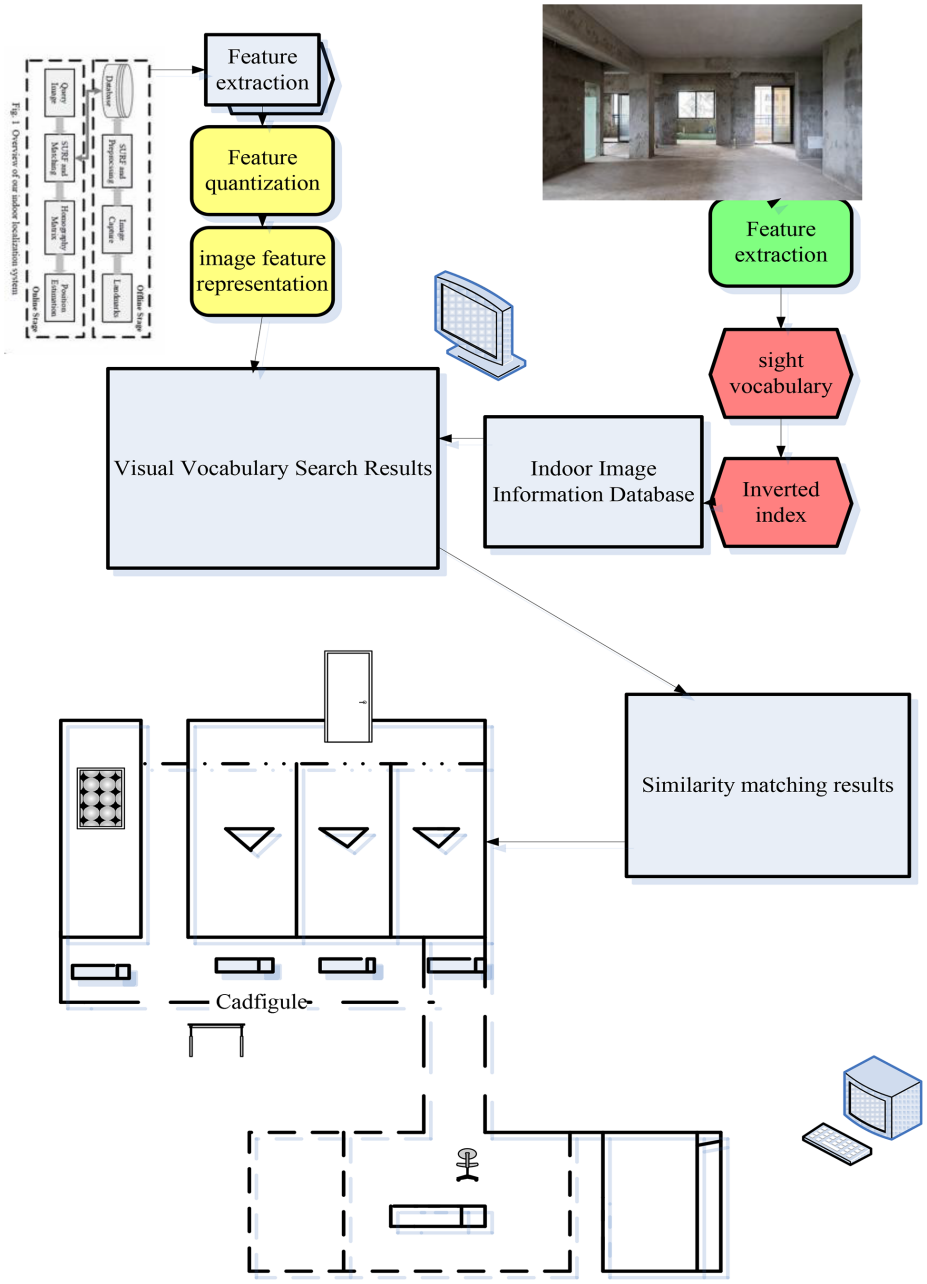
System framework and process diagram.

In this paper, an efficient vision-based indoor localization system is proposed. It uses the bag-of-words model algorithm in the image retrieval technology to extract and retrieve the image information requested for positioning, and obtain the most similar images and positions, so as to realize the positioning. SIFT, that is, scale-invariant feature transformation, is a description used in the field of image processing. This description has scale invariance and can detect key points in the image. It is a local feature descriptor. The system combines SIFT feature extraction, bag-of-words model, and location matching to search and retrieve indoor scene images. Users capture and send query images and get location feedback through their smartphones. This paper proposes a system to build a bag-of-words model based on SIFT features to obtain the visual vocabulary of indoor scenes. It establishes an inverted file index for the image data set of the indoor scene, and then performs location matching. In the image retrieval stage, the first step is to retrieve images with the same image by matching the visual vocabulary of the query image. The second step is to perform position matching. It calculates the similarity between the candidate images retrieved in the first step and the query image and ranks them. Then, it votes for the position of the top three images. If the vote is passed, the position will be returned, and if the vote is not passed, the position matching algorithm will be carried out one by one. The position matching algorithm combines the homography constraints between SIFT feature points, which ensures the accuracy of position matching. In order to ensure the simplicity of the user’s operation, the user’s client only requires the user to shoot and send the scene in which he is located, and the server returns the client’s user’s location through retrieval and matching. The main system framework and process of this paper are shown in Figure 1.

#### The Experimental Environment of This Experiment

**Figure 2:**
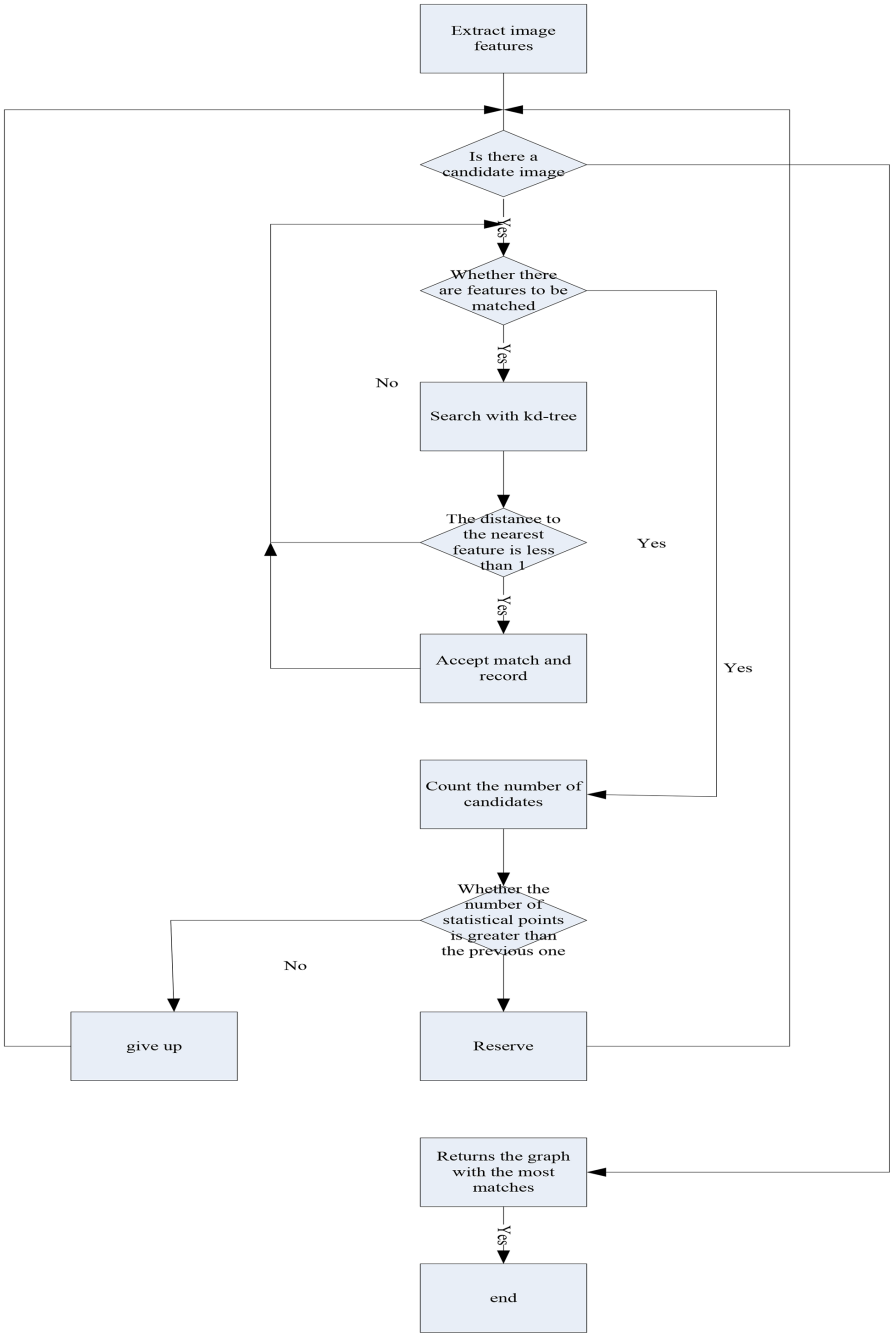
Algorithm flowchart.

Pentium(R) dual-core CPU, main frequency 3.20 GHz, memory 2 GB, operating system Windows 7, and development environment is MATLABR2008a. In order to verify the performance of the improved SIFT feature descriptor, a simple matching algorithm based on kd-tree is used. It uses an improved SIFT algorithm, which extracts SIFT feature points and SIFT feature descriptors from the training atlas and stores them in kd-tree. kd-tree is widely used in SIFT feature point matching, which can quickly and accurately find the nearest neighbors of query points. The basic idea is to divide the search space hierarchically without overlapping. It searches the training atlas for the k closest SIFT features to the SIFT features in the test images. When the distance between the closest feature point searched and the query feature point is within a certain threshold, it records the feature point as a matching feature point. When most of the features in an image are matched with the query image, the image is regarded as a matching image. The algorithm flow is shown in Figure 2:

In the experiment *k* = 1, so searching through kd-tree will return a nearest neighbor feature point. It is at a distance d from the query image feature. It only accepts this match if *d* < T1, otherwise it rejects. This paper observes the similarities and differences between various SIFT feature descriptors. In the experiment, SIFT features with thresholds T1, 128D, 96D, 64D, and 32D were determined for SIFT feature descriptors of different dimensions in image matching. The distance thresholds corresponding to the descriptors are 170, 160, 150, and 90 (Pian et al., 2017). The candidate image with the largest number of matching points is considered as the best matching image.

**Figure 3:**
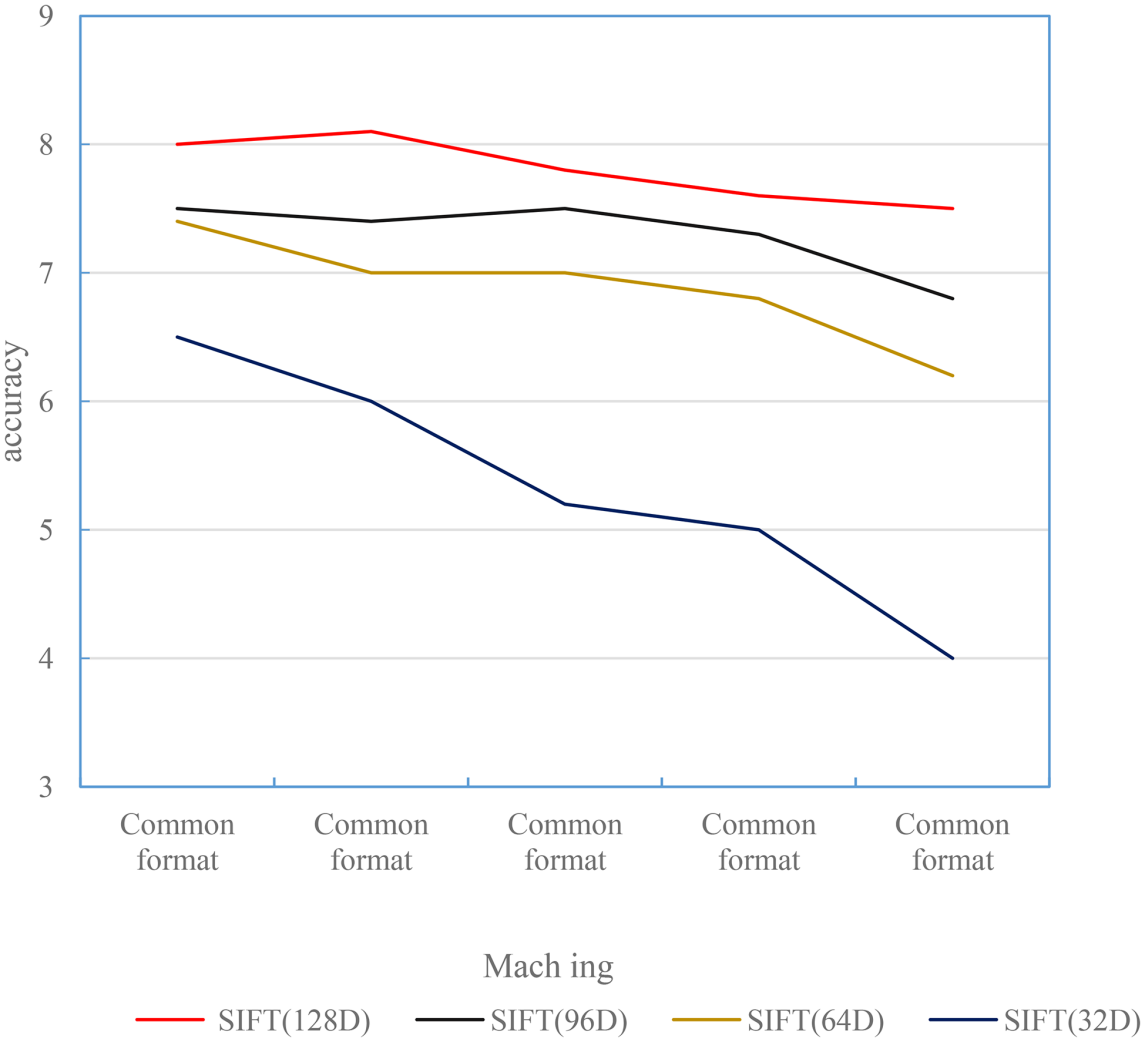
Rotated image matching accuracy.

The matching results of the rotated images are shown in Figure 3. The results show that the SIFT feature descriptors of 32D, 64D, 96D, and 128D all have good orientation invariance.

#### Experiments With Illumination Invariance

**Figure 4:**
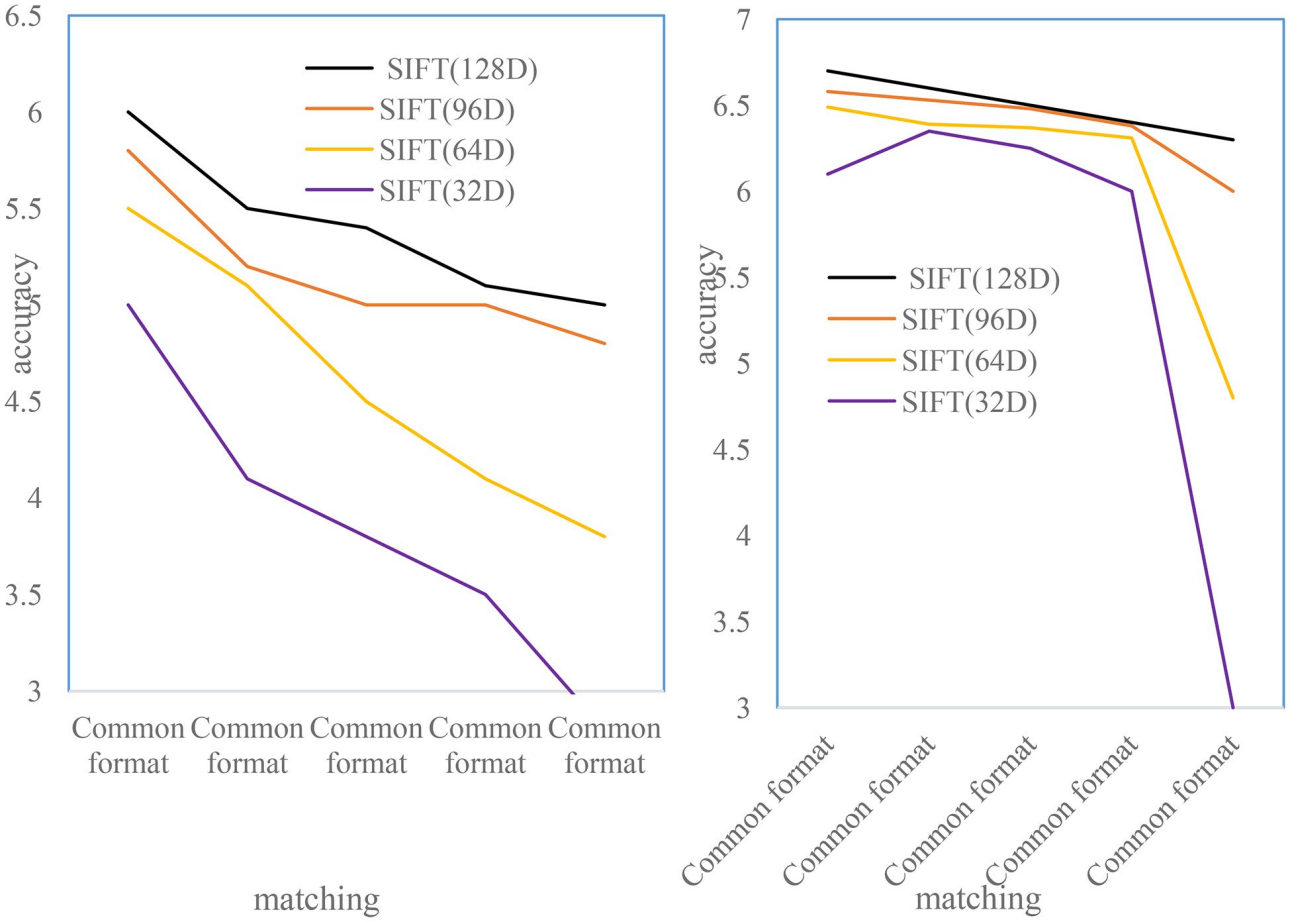
Lighting enhancement reduces matching accuracy.

To test the illumination invariance of the SIFT feature descriptor, it adds or subtracts a portion of the pixel offset from the same 500 images, such as increasing the red, green, and blue intensity of each pixel at the same time. As shown in Figure 4, the offset is generally between 0 and 255. The pixel offsets tested are 50, 70, 100, 120, −30, −50, −70, −100, and −120.

#### Noise Invariance Experiment

**Figure 5:**
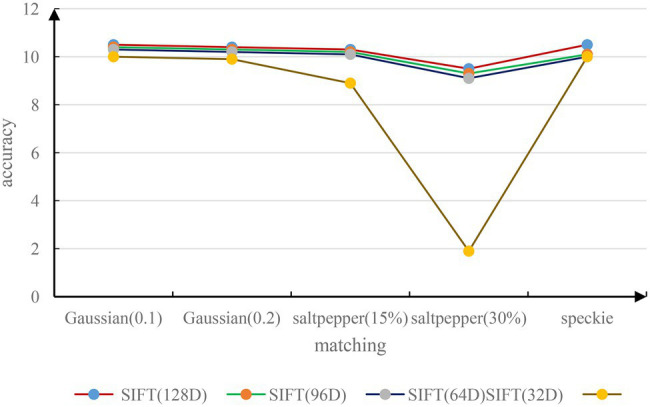
Matching accuracy of images with different noises.

To test the noise invariance of the SIFT feature descriptor, the experiment uses the imnoise function in MATLAB to add noise to the image. It uses three kinds of noise: Gaussian noise, salt and pepper noise, and speckle noise. Before adding noise, the pixels of each test image should be normalized first. Here, we use white Gaussian noise of $\phi^{2}$ = 0.1, salt and pepper noise of 15% and 30%, and a mean of 0. The experimental results of speckle noise with a variance of 0.04 are shown in Figure 5. Experiments show that the matching accuracy of SIFT feature descriptor will be significantly reduced in the environment of salt and pepper noise. This situation is more obvious for small-dimensional SIFT descriptors.

**Figure 6:**
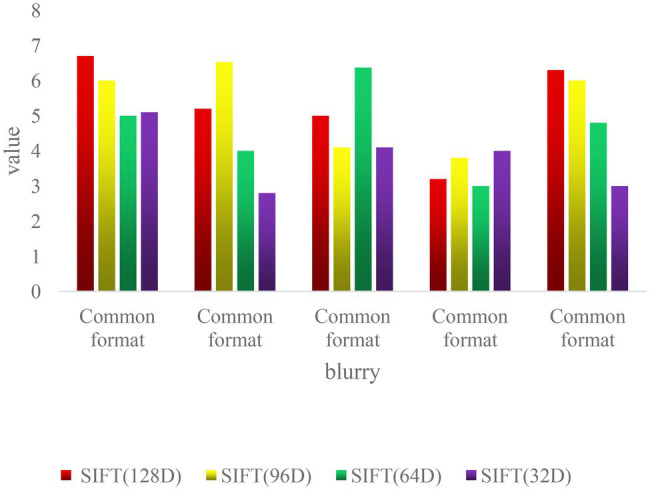
Matching accuracy of different blur levels.

As shown in Figure 6, the matching performance of SIFT feature descriptors of all scales is not affected in the case of image blur. When *σ* = 20, the test image is too blurred, which reduces the matching performance of 32D SIFT feature descriptors. In the case of high ambiguity, large-dimensional SIFT feature descriptors are less affected (Katsanos and Vamvatsikos, 2017).

#### Scale Transformation

**Figure 7:**
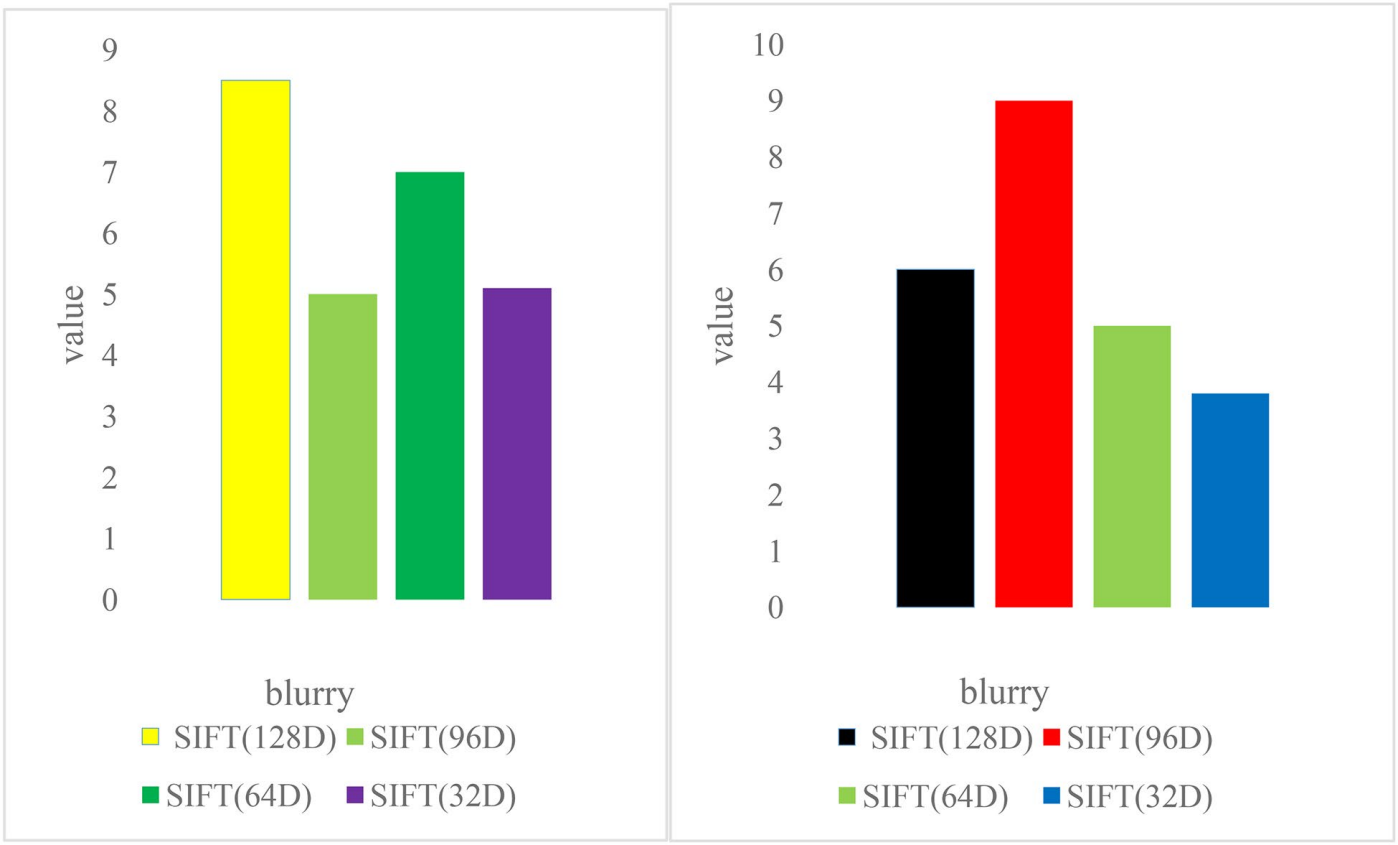
Image matching accuracy at different scales.

To test the scale invariance, 500 objects were selected from the image dataset in the experiment. Each object has two images of different scales, with a total of 1,000 images. All the selected object images have scale transformation, and some images also have perspective transformation. One image of each object is used for testing and the other is used for training. As shown in Figure 7, the 128D, 96D, and 64D SIFT feature descriptors have good matching performance in the case of scale transformation, and the 32D SIFT feature has poor matching performance. Moreover, the SIFT matching accuracy of 96D and 64D is even higher than that of the 128D SIFT feature descriptor. This is because the improved 96DSIFT and 64DSIFT make the matching center of gravity more concentrated in the vicinity of the feature points by calculating the gradient value weights of different regions.

#### Perspective Transformation

To test perspective invariance, images of 500 objects were selected from the image dataset. Each object has two images from different perspectives, with a total of 1,000 images. The perspective transformation of each object is different. The first image of the object is used for testing and the other is used for training. The experimental results are shown in Figure 7. The matching performance of all SIFT feature descriptors under perspective transformation is worse than that of other cases, and the matching accuracy of 64D SIFT feature descriptors is higher than others (Oteda et al., 2021).

### Experimental Results and Analysis

**Figure 8:**
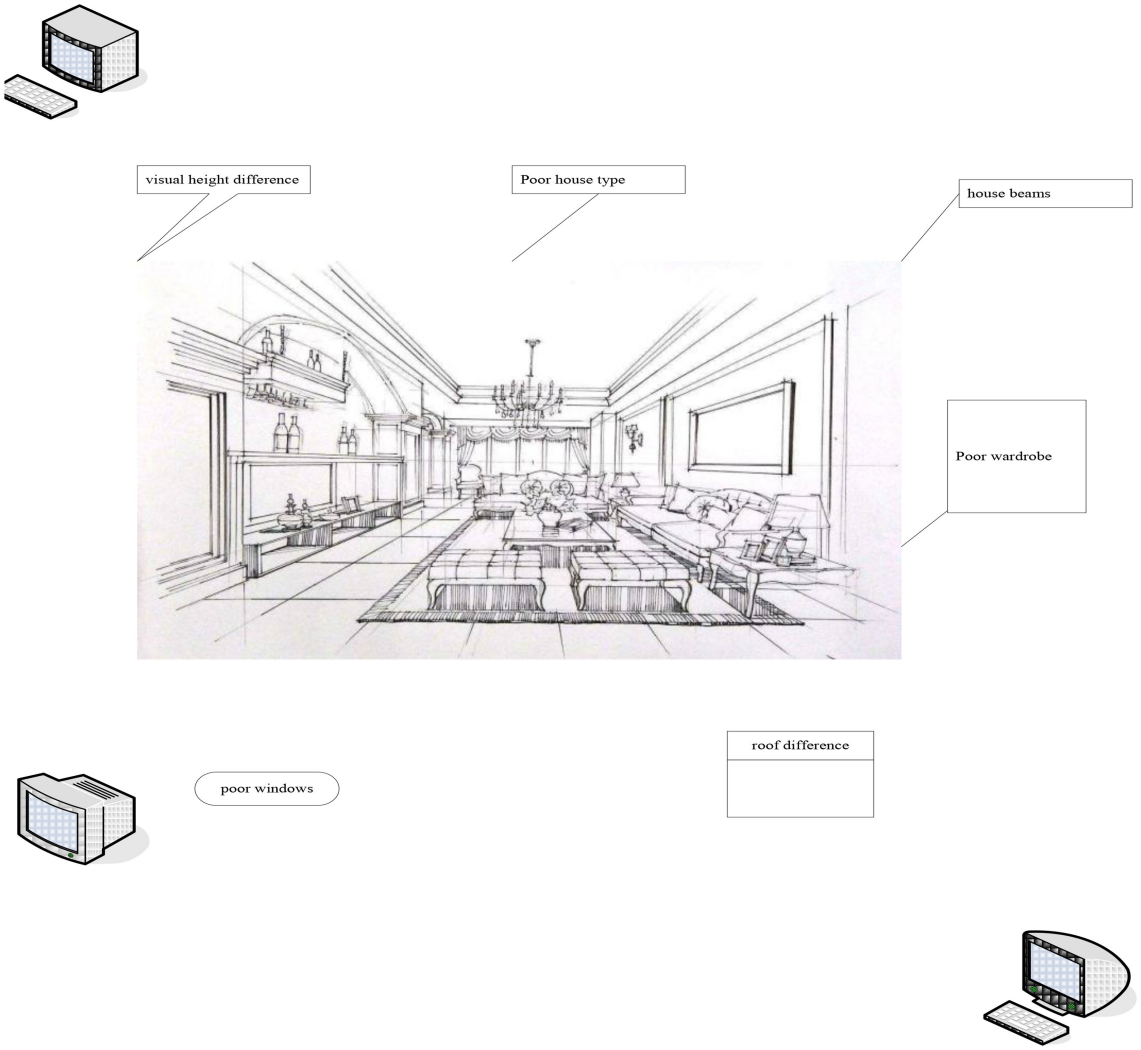
The effects of poor vision.

Through the operation of the above four different algorithms, the experimental results that can be obtained are mainly the position of the returned query image and whether the position matching algorithm is carried out in a query. The experimental results are shown in Figure 8. It will then analyze the results of the experimental summary data.

#### Matching Accuracy Analysis of the Four Algorithms

**Figure 9:**
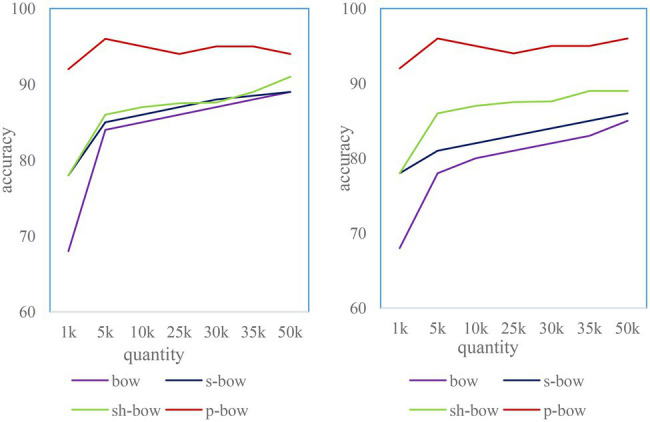
Matching accuracy of four algorithms under different number of clusters.

**Figure 10:**
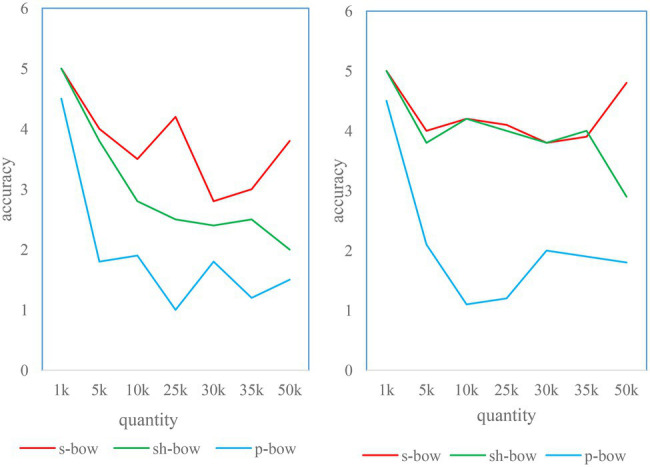
Standard deviation of three position matching algorithms under different weighting schemes.

Each algorithm was run 15 times for cross-validation. The average matching accuracy is shown in Figure 9, and the system SD is shown in Figure 10. The results show that the homography-based location confirmation algorithm performs the best in terms of matching accuracy, and also has very high stability (low SD). The matching accuracy of the location matching algorithm based on the color histogram is more stable than that of the location confirmation algorithm based on the SIFT distance. This is because of the addition of color information.

In order to further analyze the performance of the homography-based position matching algorithm, we choose to consider only the matching accuracy under non-ideal conditions in the following analysis. It serves as a metric in the following two aspects (Simpal, 2020).

1. Unable to match ratio (NoDecisionRate, R.R): the number of query images cannot get the best match through the position matching algorithm.
2. Correct matching ratio (Correctacceptancerate, C.A): it is the correct matching ratio after passing through the position matching algorithm.

## Discussion

In this paper, the use of image retrieval techniques to implement an indoor positioning system is introduced. It experimentally analyzes the effects of different locations from the perspective of visual capture. However, because the vision-based indoor positioning system needs to collect and preprocess the indoor scene images in advance, the image quality of the image database and the richness of the image database will affect the accuracy of position matching. Large-scale datasets require more algorithmic improvements to improve retrieval efficiency (Deng, 2018). First, it combines optical illusion with various information for positioning, using GPS and WiFi positioning technology to combine with the vision-based indoor positioning system proposed in this paper. This enables the localization of more indoor scenes (Huang et al., 2017; Norford, 2018). For example, it uses GPS to first locate the location of the user. When the user puts forward a positioning requirement, the system can first retrieve the image data set of the user’s location through the GPS positioning information, and then perform detailed positioning. This expands the influence of visual effects on positioning techniques in interior design.

## Conclusion

People live in a three-dimensional world, but the images perceived by the human eye are indeed two-dimensional. People are exposed to the shape, size, distance, texture, and other information of objects since childhood. Then we analyze it through the brain, because people’s understanding of space is not innate. Our brains need to touch, feel, and analyze space to fully understand this three-dimensional world. At present, indoor visual positioning technology has not appeared in relatively excellent actual product systems, and most of them are only laboratory research. Considering some practical problems encountered in the research process of this paper, the following research work will conduct more detailed research and improvement in the following directions. The development of deep learning in recent years seems to have become a research trend. Deep learning has shown good performance in all aspects, so in the next research process, more in-depth research on intelligent algorithms is required. It needs to learn from the research method of neural network algorithm to improve the scene classification and recognition rate. In terms of fusion algorithm, Kalman filter technology is used in this paper. Compared with other advanced filtering technologies, it lacks comparability, and the experimental environment is a relatively simple indoor environment. Therefore, it requires more complex experiments in follow-up work. In addition, we can consider trying to combine better filtering techniques to fuse the data and remove impurities. In the actual research process of this topic, due to the limited theoretical knowledge and the lack of practical experience, there are also some places that contradict the traditional knowledge structure and inertial thinking. The research results of the paper still need to be continuously enriched and improved. The theoretical breadth and depth of thinking are still lacking, and the shortcomings will be continuously improved and comprehended in future studies and work.

## Author’s Note

YX was born in Jiangsu, China, in 1981. She received the Master degree from Tianjin Polytechnic University, China. Now, she studies in the School of Economics and Management, NanJing University of Aeronautics and Astronautics. Her field of study is environmental design.

E-mail: yunkai@nuaa.edu.cn.

TY was born in WenZhou, Zhejiang province, China, in 1987. She received the Master degree from Kunming University of MFA, China. Now, she works in the School of architectural engineering, Wenzhou Polytechnic. Her research interests include intelligent interior design for the elderly. Research on interior design culture.

E-mail: 2021000528@wzpt.edu.cn.

## Data Availability Statement

The original contributions presented in the study are included in the article/supplementary material, further inquiries can be directed to the corresponding author.

## Author Contributions

YX: writing and static analysis of data. TY: guiding the research directions and ideas. All authors contributed to the article and approved the submitted version.

## Funding

This work was supported by the Ministry of Education Humanities and Social Sciences Research project “project approval number 20YJCZH218” under healthy Chinese perspective integrated into the intelligent elderly housing space planning design research.

## Conflict of Interest

The authors declare that the research was conducted in the absence of any commercial or financial relationships that could be construed as a potential conflict of interest.

## Publisher’s Note

All claims expressed in this article are solely those of the authors and do not necessarily represent those of their affiliated organizations, or those of the publisher, the editors and the reviewers. Any product that may be evaluated in this article, or claim that may be made by its manufacturer, is not guaranteed or endorsed by the publisher.
